# Soil bacterial community structures in relation to different oil palm management practices

**DOI:** 10.1038/s41597-020-00752-3

**Published:** 2020-11-30

**Authors:** Dirk Berkelmann, Dominik Schneider, Nina Hennings, Anja Meryandini, Rolf Daniel

**Affiliations:** 1 Genomic and Applied Microbiology and Göttingen Genomics Laboratory, Institute of Microbiology and Genetics, Georg-August-University of Göttingen, Göttingen, Germany; 2 Department of Soil Science of Temperate Ecosystems, Buesgen Institute, University of Göttingen, Göttingen, Germany; 3 Department of Biology, Faculty of Mathematics and Natural Sciences IPB, Bogor Agricultural University, Bogor, Indonesia

**Keywords:** Microbial ecology, Microbial ecology

## Abstract

We provide soil bacterial 16 S rRNA gene amplicon and geochemical data derived from an oil palm plantation management experiment. The experimental design covered two different intensities of fertilizer application and weeding practices. We sampled the topsoil of 80 plots in total and extracted DNA and RNA. 16 S rRNA gene-derived and transcript-derived amplicons were generated and sequenced to analyse community composition and beta-diversity. One year after establishing the experiment, statistically significant differences of bacterial diversity or community composition between different treatments at entire (DNA-derived) and active (RNA-derived) community level were not detected. The dominant taxa belonged to *Acidobacteriota* and *Actinobacteriota* and were more abundant in the active community compared to the entire community. Similarly, the abundant genera *Candidatus* Solibacter and *Haliangium* were more abundant at active community level. Furthermore, clustering corresponding to the different sampling site locations was detected. Beta-diversity did not change among the treatments at DNA and RNA level. This dataset is of interest for related studies on the effect of altered management practices on soilborne communities.

## Abstract (summary)

**Table:** 

Measurement(s)	DNA • RNA
Technology Type(s)	DNA sequencing • RNA sequencing
Factor Type(s)	intensity of fertilizer application • weeding practice • plot
Sample Characteristic - Environment	soil • oil palm plantation
Sample Characteristic - Location	Sumatra

Machine-accessible metadata file describing the reported data: 10.6084/m9.figshare.13181618

## Background & Summary

Palm oil is used in a variety of products, from cooking oil to biofuel with increasing global demand^1^. Thus, palm oil production has been scaled up dramatically in the last decades, leading to massive deforestation^2,3^. The biggest producer of palm oil is Indonesia, which also shows the highest rates of deforestation^4^. Due to the accompanying diversity loss, rainforest conversion and large-scale palm oil production is heavily debated and subject to research in various disciplines^5–11^. Additionally, the effects of fertilizer and herbicide applications on diversity and nutrient cycling in soil are considered as important factors for oil palm cultivation^12–15^. As bacteria mediate almost all nutrient cycling pathways in soils, several studies focused on the impact of rainforest conversion and oil palm cultivation on soilborne bacterial communities^6,16–18^. These studies showed that certain groups, which are connected to nutrient cycling pathways, are affected by rainforest conversion to managed land use systems. These comprised the proteobacterial groups *Rhizobiales* and *Burkholderiales* as well as taxa benefitting from rainforest conversion such as *Acidobacteriales*, Subgroup 2 of *Acidobacteriota* and *Streptomycetales*^5,6^. These results emphasized the effects of rainforest conversion to managed oil palm plantations, which are subjected to fertilizer, herbicide and/or mechanical weeding applications.

In this study, based on 16 S rRNA gene and transcript sequencing and analysis, we provide data regarding the effects of reduced fertilizer application and mechanical weeding practices on soil bacterial communities. The analysis was performed in a state-owned oil palm plantation in Sumatra, Indonesia. Four different treatments, consisting of combinations of conventional and reduced fertilizer application, as well as mechanical or herbicide-based weeding practices were analysed (Table 1).

**Table 1:** Applied management practices of the four treatment types, including the applied nutrient amounts per hectare and year.

Treatment ID	Treatment	Fertilizer used	Weeding (interrow)	liming
**ch**	Conventional fertilization + herbicide	260 kg N ha^−1^ yr^−1^, 50 kg P ha^−1^ yr^−1^, 220 kg K ha^−1^ yr^−1^	750 cm^3^ glyphosate ha^−1^ yr^−1^	426 kg dolomite ha^−1^ yr^−1^, 142 kg micromag ha^−1^ yr^−1^
**cw**	Conventional fertilization + mechanical weeding	260 kg N ha^−1^ yr^−1^, 50 kg P ha^−1^ yr^−1^, 220 kg K ha^−1^ yr^−1^	mechanical	426 kg dolomite ha^−1^ yr^−1^, 142 kg micromag ha^−1^ yr^−1^
**rh**	Reduced fertilization + herbicide	136 kg N ha^−1^ yr^−1^, 17 kg P ha^−1^ yr^−1^, 187 kg K ha^−1^ yr^−1^	750 cm^3^ glyphosate ha^−1^ yr^−1^	426 kg dolomite ha^−1^ yr^−1^, 142 kg micromag ha^−1^ yr^−1^
**rw**	Reduced fertilization + mechanical weeding	136 kg N ha^−1^ yr^−1^, 17 kg P ha^−1^ yr^−1^, 187 kg K ha^−1^ yr^−1^	mechanical	426 kg dolomite ha^−1^ yr^−1^, 142 kg micromag ha^−1^ yr^−1^

**Fig. 1:**
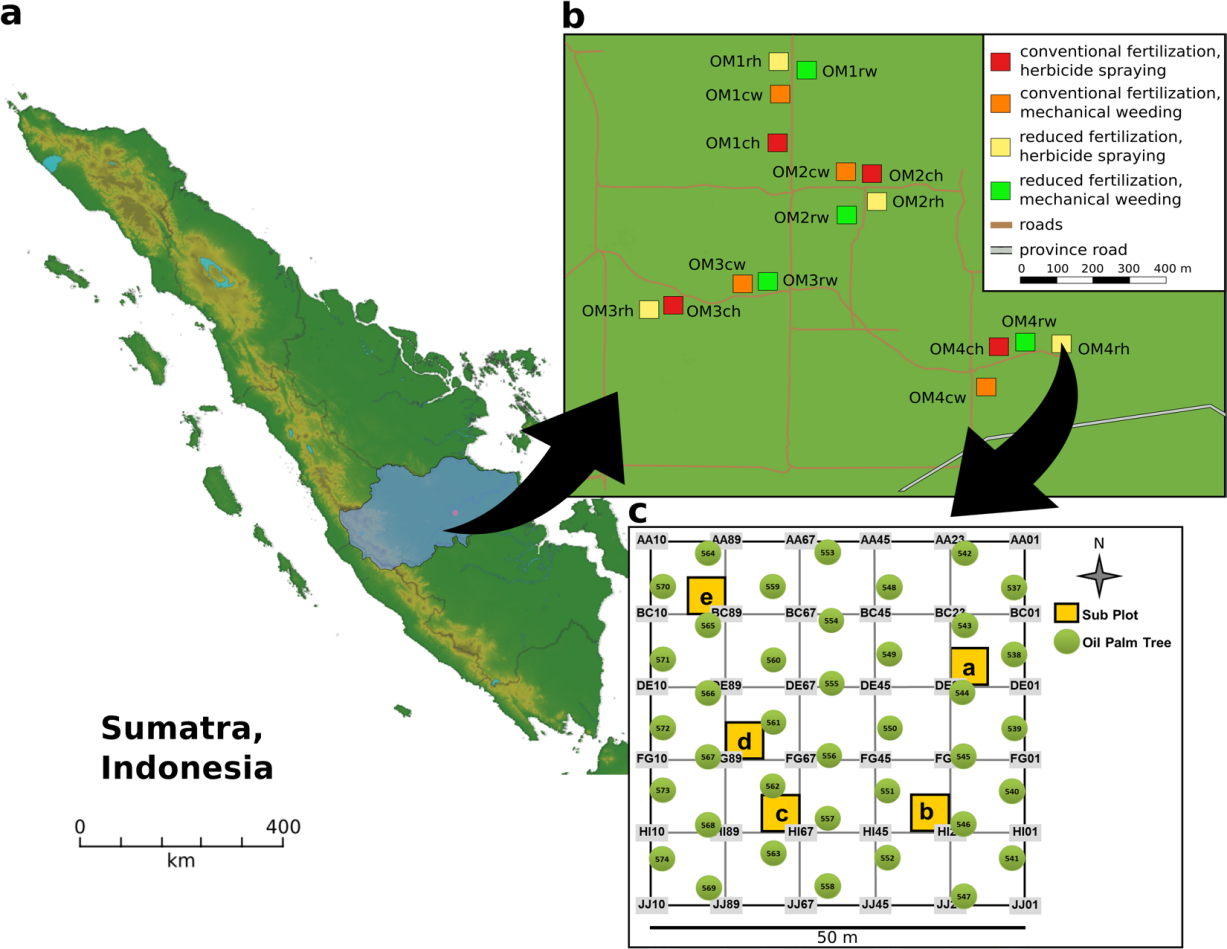
Sampling sites and experimental design of all sampling sites in Jambi, Indonesia. The location of the oil palm plantation in the province of Jambi is shown in respect to Jambi City (**a**). Squares show each plot with the respective treatment indicated by color, with four replicates per treatment (**b**). An example of the experimental design is shown for each plot (**c**), with squares a-e indicating subplots and green circles showing planted oil palm trees.

The experimental sites were established in November 2016 with four replications per treatment in short distance to each other (Fig. 1). We extracted DNA and RNA from topsoil in all plots, amplified the V3-V4 region of the 16 S rRNA genes and transcripts and sequenced the resulting amplicons. Additionally, soil attributes were measured for all soil samples to identify potential correlations with the corresponding soil bacterial community^19^.

**Fig. 2:**
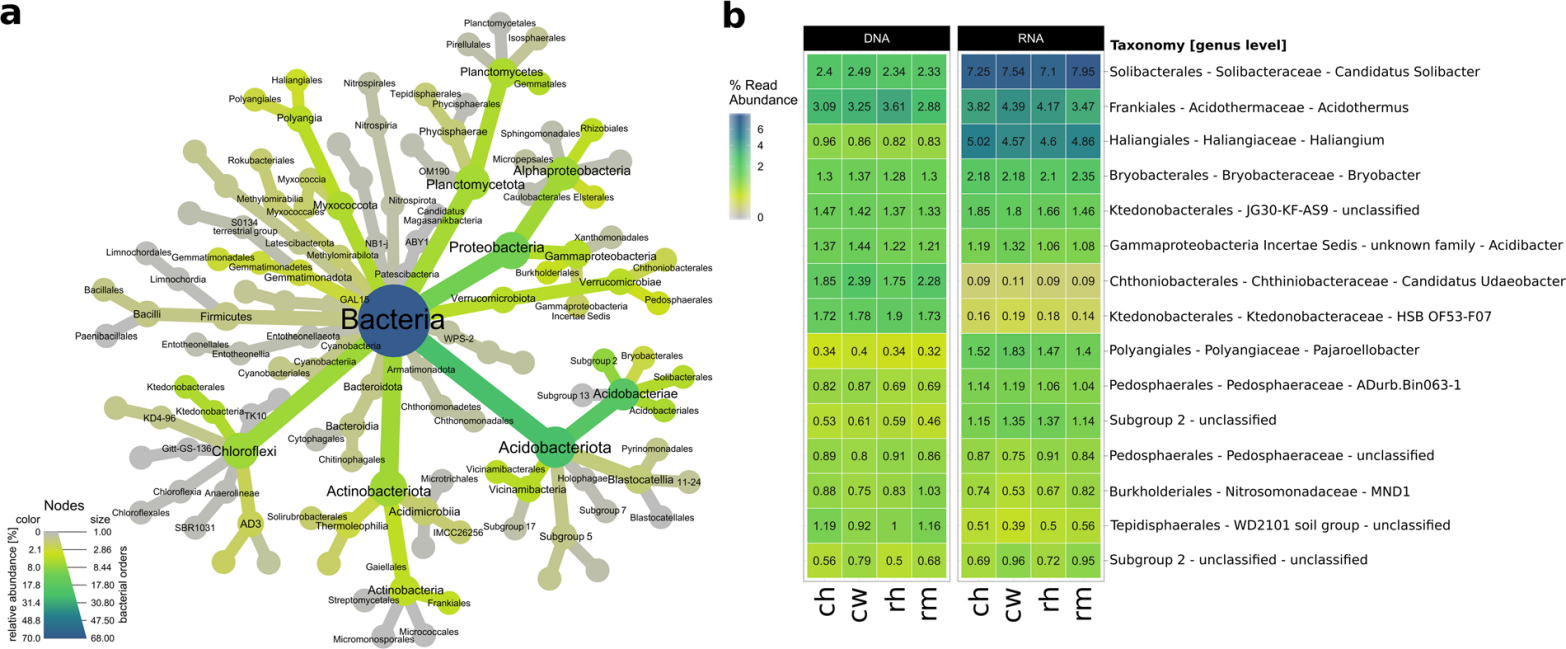
Community composition in all treatments at different taxonomic levels. The average community of the entire (DNA-based) and potential active (RNA-based) community at order level is shown as heat-tree (**a**) including all higher taxonomic levels for all used sequences. A separate visualization of the fifteen most abundant genera (**b**) is shown as relative abundances at genus level for the entire and the active community.

We analysed community composition, diversity and correlations to abiotic soil parameters. Most abundant phyla in the entire dataset were *Acidobacteriota* (formerly known as *Acidobacteria*), *Proteobacteria* and *Actinobacteriota* (formerly *Actinobacteria*) (Fig. 2a,b). At order level, *Acidobacteriota* (Subgroup 2), *Acidobacteriales* and *Ktedonobacterales* were the most abundant groups with no significant differences in relative abundance between the different treatments. At genus level, the most abundant taxa belonged to *Acidobaceriota* (*Candidatus* Solibacter and *Bryobacter*), *Actinobaceriota* (*Acidothermus*) and *Myxococcota* (*Haliangium*) (Fig. 2b). Again, we did not detect statistically significant changes between the treatments, but notable differences between the entire (DNA-based) and potentially active (RNA-based) community for some genera. Especially the abundant genera *Candidatus* Solibacter and *Haliangium* showed higher abundances in the active communities with relative abundance increases from 2.3 to 7.4% (*Candidatus* Solibacter) and 0.8 to 4.8% (*Haliangium*). In contrast, *Candidatus* Udaeobacter and HSB OF53-F07 of the *Ktedonobacterales* were among the ten most abundant genera of the entire community (approximately 2 and 1.7%, respectively) but represented only approximately 0.2% at active community level in all samples. In general, the detected bacterial community composition was similar to previously described communities in oil palm soils^5,6,20,21^, which are mostly managed in a similar fashion with respect to conventional fertilizer and herbicide treatment^5,6,21^.

**Fig. 3:**
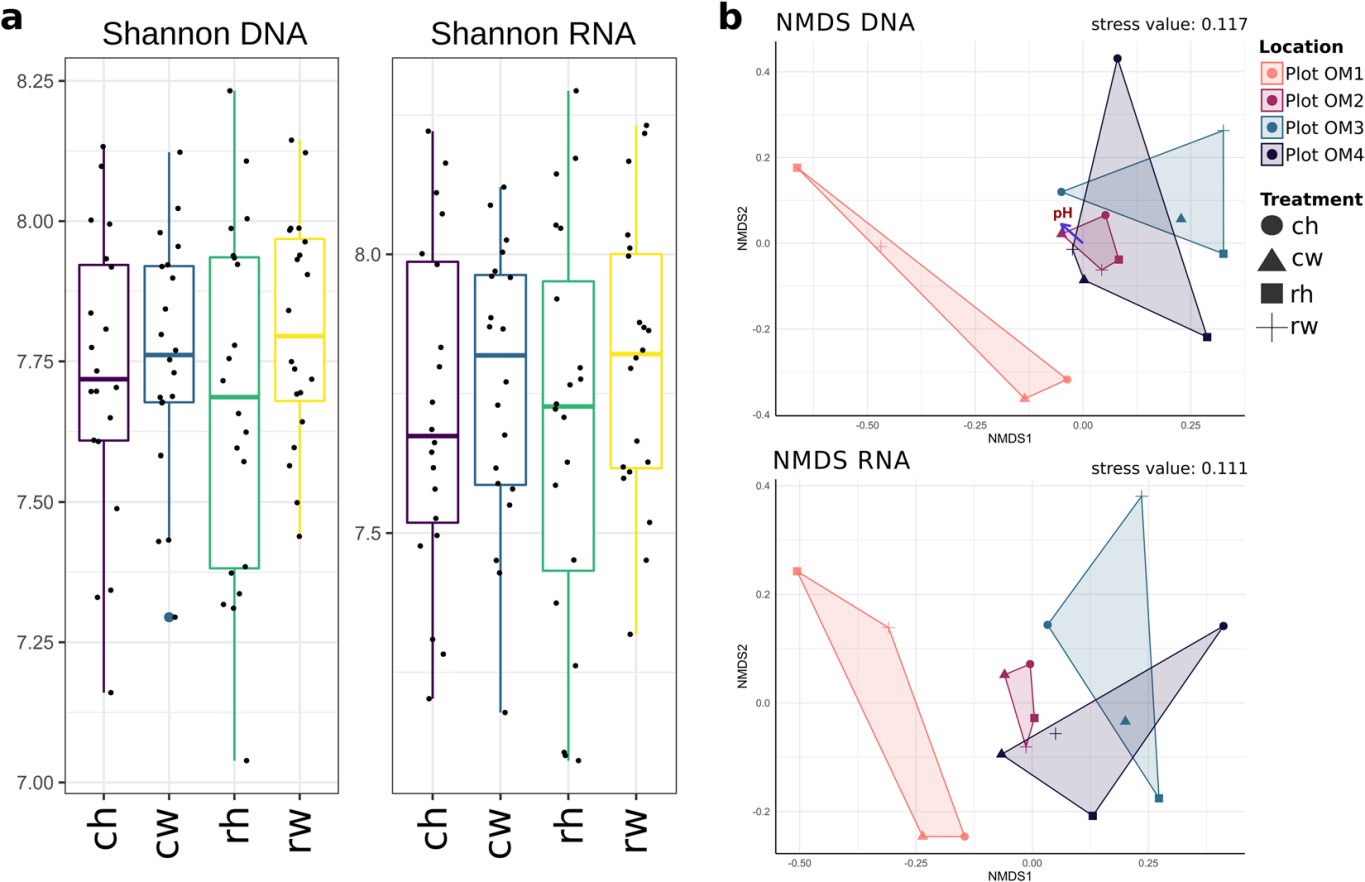
Detected diversity and multivariate analysis of all analyzed plots. Diversity is displayed by the Shannon index (**a**) for DNA- and RNA-derived sequences. Non-metric multidimensional scaling (NMDS) is shown for DNA- and RNA-derived sequences (**b**). The samples were clustered at plot level, with frames and colors showing the four different plot locations and shapes for the different treatments. Significant correlations with abiotic measurements are indicated by purple arrows.

We also analysed the diversity at potential active and entire community level by using the Shannon index. Values ranged from 7.0 to 8.2 with no significant changes between the different treatments (Fig. 3a). Ordination analysis by NMDS did not show distinct clustering according to treatments but clustering due to geographical location was observed, emphasizing the importance of location over treatment. (Fig. 3b). Furthermore, we detected a significant correlation between the bacterial community at DNA level and pH (p < 0.015, R^2^ = 0.45). No correlations were detected with other measured abiotic factors (p > 0.05).

## Methods

### Site description and soil sampling

The experiment was established as part of the EFForTS project (**E**cological and socioeconomic **F**unctions of tropical lowland rain**For**est **T**ransformation **S**ystems) in the Jambi province, located in Sumatra, Indonesia^8^.

The experimental sites are located in the state-owned oil palm plantation PTPNVI, which was planted in 2002 (Fig. 1). All planted palms were derived from Tenera seedlings, which are a crossing between Dura and Psifera palms, supplied by Marihat (Medan, Indonesia). Four different locations (referred to as OM1-4) harbor four treatments, which were established in November 2016. In each of these 16 plots (50 × 50 m), five subplots were randomly established, resulting in 80 samples total.

Fertilizer treatment was conducted in two intensities: for one application the conventional treatment usually used in the entire plantation with 130 kg nitrogen, 25 kg phosphorus and 110 kg potassium ha^−1^ and reduced fertilization with 68 kg nitrogen, 8.5 kg phosphorous and 93.5 kg potassium ha^−1^. Additionally, liming was conducted in all plots with equal amounts (213 kg dolomite and 71 kg micromag (micronutrients) ha^−1^). Fertilizer application and liming was done twice per year. The herbicide treatment used 375 cm^3^ glyphosate ha^−1^ sprayed within the palm circle four times per year and 375 cm^3^ glyphosate ha^−1^ in inter-rows applied twice per year^15^. The last application before sampling was done in April 2017. Mechanical weeding was done by cutting vegetation four times per year within the palm circle and two times per year in interrows with a brush cutter. The combination of these applications resulted in four different treatments: conventional fertilization with herbicide spraying (ch), conventional fertilization with mechanical weeding (cw), reduced fertilization with herbicide spraying (rh) and reduced fertilization with mechanical weeding (rw) (Table 1).

Topsoil was sampled in May 2017 with a soil corer from the upper seven centimeters in each subplot with a diameter of five cm. A soil corer was used to take three cores in each subplot with a distance of 1 m to each other and at least 1 m distance to trees. The three bulk soil samples per subplot were homogenized and coarse roots and stones were removed. To prevent nucleic acids, especially RNA, from degradation RNAprotect Bacteria Reagent (Qiagen, Hilden, Germany) was applied in a ratio of 1:1. For measurements of soil parameters, we collected an additional sample, which was not supplemented with RNAprotect solution. All samples were transported in cooling boxes and stored at −80 °C until further use.

### Nucleic acid extraction

Frozen samples were thawed on ice. RNAprotect was removed from all samples by centrifuging for 20 min at 804.96 *g* and 4 °C and discarding the resulting supernatant. DNA and RNA were co-extracted from 1 g of soil by using the Qiagen RNeasy PowerSoil Total RNA kit and the RNeasy PowerSoil DNA Elution kit as recommended by the manufacturer (Qiagen), except that RNA was eluted with 50 µl elution buffer instead of 100 µl. DNA contamination was removed from RNA preparations by using the TurboDNAfree kit (Applied Biosystems, Darmstadt, Germany). For this purpose, 0.1 volume DNAse buffer and 1 µl DNAse were added and incubated for 30 min at 37 °C. Subsequently, a second digestion cycle was performed with 0.5 µl DNAse at 37 °C for 15 min. RNA was then purified with the RNeasy MiniElute Cleanup kit (Qiagen). In order to verify complete DNA removal, a control amplification of the 16 S rRNA gene was performed as described below for 16 S rRNA gene amplification. Purified RNA was then reverse-transcribed into cDNA with the Superscript IV reverse transcriptase and a specific primer (5′-CCGTCAATTCMTTTGAGT-′3) as recommended by the manufacturer (Thermo Fisher Scientific, Schwerte, Germany). After cDNA synthesis, we removed residual RNA by adding 1 µl RNase H (New England Biolabs, Frankfurt am Main, Germany) to each reaction and incubation for 20 min at 37 °C. Obtained DNA and cDNA were stored at −20 °C until further use.

### 16 S rRNA gene amplification and sequencing

For amplification of 16 S rRNA sequences, we used 16 S rRNA gene primers targeting the V3-V4 region (forward primer: S-D-Bact-0341-b-S-17 5′-TCGTCGGCAGCGTCAGATGTGTATAAGAGACAG-CCTACGGGNGGCWGCAG-3′, reverse primer: S-D-Bact-0785-a-A-21 5′-GTCTCGTGGGCTCGGAGATGTGTATAAGAGACAG-GACTACHVGGGTATCTAATCC-3′) as described by Klindworth^22^ and Herlemann^23^ and added adapters for MiSeq sequencing (underlined). PCR reactions were performed in a total volume 50 µl containing 10 µl of 5-fold Phusion GC buffer, 0.2 µl 50 mM MgCl_2_ solution, 2.5 µl DMSO, 200 µM of each of the four deoxynucleoside triphosphates and 1 U of Phusion High-Fidelity DNA Polymerase (Thermo Fisher Scientific). We used 20 to 30 ng of DNA and 1 µl cDNA per reaction. The PCR reaction was started by an initial denaturation at 98 °C for 1 min, followed by 25 cycles of denaturation at 98 °C for 45 s, annealing at 60 °C for 45 s and elongation at 72 °C for 30 s. The final elongation was at 72 °C for 5 minutes. Amplicons were then purified by using MagSi-NGS PREP Plus magnetic beads following the procedure recommended by the manufacturer (Steinbrenner Laborsysteme GmbH, Wiesenbach, Germany) with the Janus Automated Workstation from Perkin Elmer (Perkin Elmer, Waltham Massachusetts, USA). Illumina MiSeq sequencing adapters were attached to the purified amplicons with the Nextera XT Index kit (Illumina, San Diego, USA). The Index PCR was done by using 5 µl of template PCR product, 2.5 µl of each index primer, 12.5 µl of 2x KAPA HiFi HotStart ReadyMix and 2.5 µl PCR grade water. Thermal cycling scheme was as follows: 95 °C for 3 min, 8 cycles of 30 s at 95 °C, 30 s at 55 °C and 30 s at 72 °C and a final extension at 72 °C for 5 min. The indexed products were purified as described before. Products were quantified by using the Quant-iT dsDNA HS assay kit and a Qubit fluorometer following the instructions of the manufacturer (Invitrogen GmbH, Karlsruhe, Germany). Purified amplicons were sequenced by the Göttingen Genomics Laboratory with a MiSeq instrument with a read length of 2 × 300 bp using dual indexing and reagent kit v3 (600 cycles) as recommended by the manufacturer (Illumina).

### Sequence processing

We obtained 6,817,019 amplicon sequences with 5,183,993 remaining sequences after quality-filtering from DNA samples. At RNA level 6,412,838 raw sequences with 3,601,637 remaining sequences after quality-filtering were obtained^24^.

Obtained paired-end sequences were first quality-filtered with fastp version 0.20^25^ using a minimum phred score of 20, a minimum length of 50 bases, the default sliding window size (–cut_window_size = 4), read correction by overlap (option “correction”), adapter removal of the sequencing primers (option “adapter_fasta”), and the provided index sequences of Illumina. Quality-filtered paired-end reads were merged with PEAR version 0.9.11 and default settings^26^. Primer sequences were clipped with cutadapt version 2.5 and default settings^27^. All further steps, except mapping of sequences to ASVs (Amplicon Sequence Variant) were performed with functions implemented in vsearch version 2.1.4.1^28^. Sequences were filtered by size with “sortbylength” with a set minimum length of 300 bp. Dereplication of identical sequences was done by “derep_fulllength”. Denoising and removal of low abundant sequences with less than eight replicates were done with the vsearch UNOISE3 module “cluster_unoise”. Chimeric sequences were removed by employing the UCHIME module of vsearch. This included a *de novo* chimera removal (“uchime3_denovo”) and a reference-based chimera removal (“uchime_ref”) against the SILVA SSU 138 NR database^29^. Sequences were mapped to ASVs by vsearch (“usearch_global”) with a set sequence identity threshold of 0.97. Taxonomy assignments were performed with BLASTN^30^ (version 2.9.0) against the SILVA SSU 138 NR database^29^ with an minimum identity threshold of 90%^31^. In addition to the taxonomy identity, we added the taxonomy id of the database, length of fragment, query percentage identity, query coverage and e-value in the taxonomy string of the table. We used identity (pident) and query coverage (qcovs) per ASV of the blast output to exclude uncertain blast hits. As recommended by the SILVA ribosomal RNA database project^32^, we removed the taxonomic assignment for blast hits if dividing the sum of percent identity and percent query coverage by 2 resulted in ≤93%. In total, 31,987 ASVs were used for downstream analysis.

### Bacterial community analysis

The bacterial community composition was further analysed in R^33^ (version 3.6.1) and RStudio^34^ (version 1.1.463). ASV counts were normalized by using the Geometric Mean of Pairwise Ratios (GMPR) of the GMPR package version 0.1.3^35^. Community compositions were then analysed by the ampvis2 package version 2.4.11 and “amp_heatmap” at genus level^36^. The fifteen most abundant genera were displayed as relative abundance and clustered at treatment level. Heat-trees were displayed by the metacoder^37^ package (version 0.3.2.9001).

For heat-tree calculation all counts were summed at order level and all taxa with a relative abundance of <1% in all samples were excluded. The average abundance of all included taxa was calculated from the rowmeans of the metacoder object and then added to the same metacoder object before plotting the heat-tree.

For diversity and ordination analysis, we used rarefaction by “amp_subset_samples” by ampvis2 as normalization of the original ASV count table. We used the Shannon diversity index as calculated by ampvis2 (“amp_alphadiv”) for diversity analysis. Differences between treatments were analysed by the vegan package. First, normal distribution was tested by shapiro.test and differences between the treatments by anova (“aov”) or Kruskal-Wallis Test (kruskal.test). Non-metric multidimensional scaling (NMDS) analysis was done with “amp_ordinate” based on Bray Curtis dissimilarity matrices. The environmental fit was calculated using the vegan package^38^ with a significance threshold of p ≤ 0.05.

Differences in the total community composition were calculated by first calculating a Bray Curtis dissimilarity matrix in R with the vegan package and then using pairwise permanova analysis with a strata flag for plot Location with “pairwise.perm.manova” of the RVAideMemoire package.

Differential abundance analysis of single taxa regarding different treatments was performed at genus and order level with the ANCOM package in R (version 2.1)^39^, with an alpha threshold of 0.05, W-statistic threshold of 0.8 and Benjamini and Hochberg P adjustment.

### Soil attribute measurements

For all abiotic measurements, soil samples were dried at 40 °C for at least 10 days. We measured pH by adding the 2.25-fold volume distilled water to at least 5 g dried soil and incubate for at least 1 h prior to measurement. For C and N content determination, root fragments were manually removed, the soil was passed through a 2 mm sieve to obtain the fine soil fraction, which was ground in a ball mill (MM200, Retsch, Haan, Germany). Depending on the expected C and N contents, 5 g of soil were weighed into tin capsules. Measurements were performed by the CN analyzer vario EL cube (Elementar Analysensysteme, Hanau, Germany). Samples were combusted at 950 °C after addition of oxygen with copper oxide as the catalyst and helium as the carrier gas. NOx gases were reduced to N_2_. CO_2_ and N_2_ were separated by an adsorption column and were detected by a thermal conductivity detector (TCD). External certified standards of plant and soil material (IVA Analysentechnik, Meerbusch, Germany) were measured as samples for calibration validation. To account for daily variation of the room conditions and check for drifts, daily factors were determined.

Na, K, Ca, Mg, Mn, Fe, Al, S and P were measured by using an iCAP 7400 ICP-OES DUO analyser (Thermo Fisher Scientific) and standard solutions for each analysed element (Bernd Kraft GmbH, Duisburg, Germany). Prior to measurements, 50 mg dried soil of each sample was lysed with 2 ml 65% nitric acid at 160 °C for 12 h, filtered and the volume adjusted to 25 ml with water.

## Data Records

All obtained sequences are available at the National Center for Biotechnology Information under the Bioproject accession number PRJNA599149 and Sequence Read Archive (SRA) accession number SRP239591, containing all 160 read archives (80 DNA samples and 80 RNA samples) as compressed fastq files^40^. The following files have been deposited in a figshare collection^41^ and can be also accessed separately: Details regarding quality-filtering and read statistics before, during and after bioinformatic processing^24^; ASV count table with taxonomic assignments^31^; metadata information for each sample along with abiotic soil measurements^19^.

## Technical Validation

The sample from each subplot was derived from three different soil cores. The PCR reactions for amplification of the 16 S rRNA genes transcripts and genes were performed in three technical replicates per sample. Negative controls without DNA or cDNA template were also performed. Correct amplicon size was determined on a 0.8% agarose gel. PCR triplicates per sample were pooled in equimolar amounts for amplicon sequencing to minimize possible PCR bias.
